# Risk of Infective Endocarditis in *Streptococcus mitis* Bloodstream Infections Among Patients with Neutropenia from Hematologic Malignancies

**DOI:** 10.1093/ofid/ofae063

**Published:** 2024-02-20

**Authors:** Miranda Monk, Nikitha R Patel, Ramy Elshaboury, David W Kubiak, Sarah P Hammond

**Affiliations:** Department of Pharmacy, Massachusetts General Hospital, Boston, Massachusetts, USA; Department of Pharmacy, Massachusetts General Hospital, Boston, Massachusetts, USA; Department of Pharmacy, Massachusetts General Hospital, Boston, Massachusetts, USA; Department of Pharmacy, Brigham and Women’s Hospital, Boston, Massachusetts, USA; Divisions of Infectious Diseases and Hematology/Oncology, Massachusetts General Hospital, Boston, Massachusetts, USA; Dana-Farber Cancer Institute, Boston, Massachusetts, USA

**Keywords:** bloodstream infection, endocarditis, hematologic malignancy, neutropenia, *Streptococcus mitis*

## Abstract

*Streptococcus mitis* commonly causes bloodstream infections (BSIs) in neutropenic patients but infrequently results in infective endocarditis (IE) in this population. Among 210 patients with neutropenia and *S. mitis* BSI, 55% underwent cardiac imaging. None were diagnosed with *S. mitis* IE; 3 had recurrent *S. mitis* BSI within 12 weeks.

## Graphical Abstract

**Figure:**
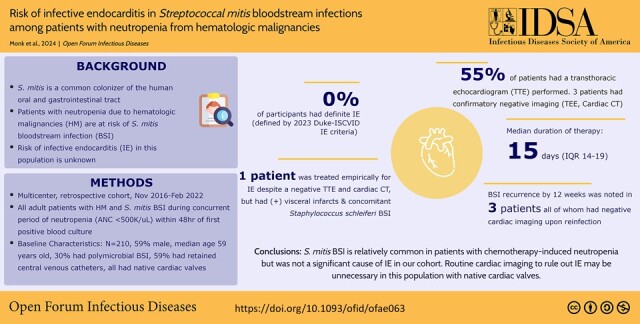
https://tidbitapp.io/tidbits/risk-of-infective-endocarditis-in-streptococcal-mitis-bloodstream-infections-among-patients-with-neutropenia-from-hematologic-malignancies-c6f48c6b-d741-4f85-b4dd-fd90b09f3d55?utm_campaign=tidbitlinkshare&utm_source=IO

*Streptococcus mitis*, an alpha hemolytic *Streptococcus* sp., is a relatively common cause of streptococcal bloodstream infection (BSI) associated with a subacute presentation of infective endocarditis (IE) in immunocompetent patients. In a recent health registry study, *S. mitis/oralis* accounted for ∼6% of monomicrobial streptococcal BSIs, but caused 19% of IE cases [1]. In a US study of streptococcal IE, alpha hemolytic *Streptococcus* was responsible for over three-quarters of cases, of which *S. mitis* accounted for 61% of alpha hemolytic *Streptococcus* IE cases [2].

Alpha hemolytic streptococci and specifically *S. mitis* are also a common cause of BSI during chemotherapy-induced neutropenia, where they cause an acute clinical syndrome, not a subacute illness. While historically BSI during neutropenia has predominately been caused by gram-negative pathogens, decades ago there was an epidemiologic shift toward gram-positive organisms including alpha hemolytic streptococci [3]. Studies of alpha hemolytic streptococcal BSI during neutropenia in the 1980s–1990s defined risk factors including mucositis, use of cytarabine chemotherapy, and use of certain antimicrobial prophylaxis agents including fluroquinolones [3, 4]. *S. mitis* is the predominate cause of alpha hemolytic streptococcal BSI during neutropenia and has been linked to an acute alpha streptococcal shock syndrome with rash, prolonged fevers, and acute respiratory distress syndrome [4, 5].

Unlike *S. mitis* BSI in immunocompetent patients where a subacute presentation of IE is a major clinical manifestation, in neutropenic hosts this pathogen behaves differently. Limited data suggest that *S. mitis* does not cause IE in neutropenic patients with hematologic malignancy (HM). In a study of 121 episodes of alpha hemolytic streptococcal BSI, 30 of 47 episodes in the general population fulfilled guideline-defined criteria for definite IE, while none of 74 episodes in patients with HM (69% of which were due to *S. mitis*) fulfilled definite IE criteria [5]. More recently, a metanalysis of patients with neutropenia and alpha hemolytic streptococcal BSI that included 35 studies (943 patients) suggested that the rate of endocarditis in the population is <1%. However, this metanalysis was limited by a lack of systematic assessment for IE (ie, cardiac imaging) in all included studies [6]. The authors suggest that routine screening for IE with either transthoracic (TTE) or transesophageal echocardiogram (TEE) may be unnecessary in this population in the absence of a new murmur.

We undertook this study to describe the incidence of IE in a contemporary cohort of neutropenic HM patients with *S. mitis* BSI at 3 institutions where cardiac imaging is pursued about half of the time. A goal of this analysis was to assess the clinical impact of cardiac imaging in this patient population.

## METHODS

We performed a retrospective cohort study of all adults with HM and *S. mitis* BSI during a concurrent period of neutropenia between November 2016 and February 2022 at Brigham and Women's Hospital, Massachusetts General Hospital, and Dana-Farber Cancer Institute, major cancer referral centers in the Northeast United States. Patient follow-up concluded on May 30, 2022. This study was approved by the Dana-Farber/Harvard Cancer Center Office for Human Research Studies.

Patients were identified by electronic medical record search for blood cultures positive for *S. mitis* during the study period. Hematologic malignancy included patients with acute and chronic leukemia, lymphoma, and multiple myeloma. Neutropenia was defined as absolute neutrophil count [ANC] <500 K/µL; patients were included if they had an ANC <500 within 48 hours of first positive blood culture.

Medical records were reviewed for covariates of interest including demographics, HM type, previous hematopoietic cell transplant, use of fluoroquinolone prophylaxis within 24 hours of bacteremia, baseline cardiac valvular conditions or cardiac devices, personal history of IE, presence and management of indwelling central venous catheters (CVCs), relevant microbiological information, and cardiac imaging results including TTE at baseline and TTE, TEE, and cardiac computed tomography (CT) within 7 days of BSI.

The primary outcome was number of patients diagnosed with IE based on updated Duke International Society for Cardiovascular Infectious Diseases (ISCVID) criteria [7]. Secondary outcomes included number of patients with findings on cardiac imaging, duration of therapy, and BSI recurrence within 12 weeks.

## RESULTS

Among 210 patients with neutropenia and concurrent *S. mitis* BSI, 123 (59%) were male, and the median age (interquartile range [IQR]) was 59 (43–66) years. Baseline demographics, microbiological information, and outcomes are shown in Table 1. Most patients had an acute febrile illness—only 3 (1%) had fever for >1 day before blood cultures were positive. All patients had native cardiac valves, and 3 (1%) had cardiac devices. Of 177 patients who had a baseline echocardiogram, 5 had moderate or severe aortic or mitral valve abnormalities (stenosis or regurgitation). Eighty-three patients (40%) had a levofloxacin-intermediate or resistant *S. mitis* isolate, of whom 53 (64%) received a fluoroquinolone within 24 hours before positive blood culture. Additionally, 62 patients (30%) had polymicrobial BSI; common co-pathogens are shown in Table 1. Most patients (n = 115, 59%) had and retained a CVC. The median duration of antimicrobial therapy for *S. mitis* BSI (IQR) was 15 (14–19) days, and 16 patients (8%) died during index hospital admission.

**Table 1.** Clinical characteristics of neutropenic patients with hematologic malignancy and *Streptococcus mitis* bloodstream infection

Baseline characteristics	n = 210, No. (%)
Median age [IQR], y	59 [43–66]
Male sex	124 (59)
Cancer type	
Acute leukemia/myelodysplastic syndrome^a^	163 (78)
Lymphoma	33 (16)
Multiple myeloma	11 (5)
Chronic leukemia	3 (1)
HCT before first positive blood culture	121 (58)
Type of HCT	
Autologous	28 (23)
Allogeneic	93 (77)
History of IE	2 (1)
Baseline TTE moderate or severe abnormality^b^	
Prosthetic valve	0 (0)
Aortic valve (stenosis [n = 3], regurgitation [n = 1], bicuspid valve [n = 1])	5 (2)
Mitral valve (regurgitation, n = 1)	1 (0.5)
Pacemaker or intracardiac device	3 (1)
FQ prophylaxis within 24 h of positive blood culture	66 (31)
qSOFA, median [IQR]	0 [0–1]
Fever (>37.9°C) at diagnosis^c^	
Fever for ≤24 h at positive blood culture	202 (98)
Fever for >24 h at positive blood culture	3 (1)
Median days of fever before positive blood culture [IQR]^c^	0 [0–0]
Microbiology	
Polymicrobial (some patients had >1 concomitant pathogen)^d^	62 (30)
Gram-negative	26
Other *Streptococcus* spp.	17
CoNS spp.	11
Anaerobes	6
Coagulase-positive *Staphylococcus* spp.	4
Other	8
≥2 bottles positive on day 1 of positive blood culture	159 (76)
Blood cultures remained positive after day 1^e^	44 (21)
Penicillin intermediate or resistant	76 (36)
Ceftriaxone intermediate or resistant	12 (6)
Levofloxacin intermediate or resistant	83 (40)
FQ exposure within 90 d of initial blood culture	68 (82)
FQ exposure within 24 h of initial blood culture	53 (64)
Clinical outcomes	
Definitive endocarditis	0 (0)
Treated empirically for possible endocarditis	1 (0.5)
Median HANDOC score^f^ [IQR]	2 [1–2]
Transthoracic echocardiogram performed	115 (55)
Median duration of therapy [IQR]^g^	15 [14–19]
Bloodstream infection recurrence within 12 wk of first positive culture^g^	3 (2)
Central line management^g^	
Removed	42 (22)
Retained	115 (59)
Retained with adjunctive antibiotic lock therapy	27 (14)
Central line not present when initial blood culture obtained	10 (5)
Died during index hospital admission	16 (8)
Positive *S. mitis* blood cultures on day of death^e^	0 (0)

Abbreviations: CONS, coagulase-negative *Staphylococcus*; FQ, fluoroquinolone; HCT, hematopoietic stem cell transplantation; IE, infective endocarditis; IQR, interquartile range; qSOFA, quick Sequential Organ Failure Assessment; TTE, transthoracic echocardiogram.

^a^Includes chronic myelomonocytic leukemia, acute myeloid leukemia, acute lymphocytic leukemia, myelodysplastic syndrome, myelofibrosis.

^b^Not all patients had baseline echocardiograms, n = 177. One patient had 2 abnormalities.

^c^n = 205; 5 patients did not have fever associated with *S. mitis* BSI.

^d^Polymicrobial blood culture:

• Other Streptococcus spp. (S. salivarius n = 9, S. parasanguinis n = 2, S. anginosus, S. gordonii, S. sanguinis, S. vestibularis, beta-hemolytic streptococci, Granulicatella adiacens n = 1).

• CoNS: (CoNS unspecified n = 5, S. epidermidis n = 4, S. capitis, S. schleiferi n = 1).

• Gram negatives (Escherichia coli n = 12, Klebsiella pneumoniae n = 9, Enterobacter cloacae n = 2, Capnocytophaga spp. n = 2, Pseudomonas aeruginosa n = 1).

• Anaerobes (Clostridium ramosum n = 2, Leptotrichia spp. n = 2, Gemella spp. n = 2).

• Coagulase-positive staphylococci spp. (methicillin-resistant S. aureus n = 1, methicillin-sensitive S. aureus n = 2, Staphylococcus intermedius n = 1).

• Other (Rothia mucilaginosa n = 3, Bacillus spp. N = 2, Enterococcus faecalis n = 1, Enterococcus faecium n = 1, identified gram-positive rod n = 1).

^e^One patient who died during the index hospitalization did not have any repeat blood cultures after the initial set, and not all patients had repeat cultures taken the day after the first positive culture.

^f^HANDOC score includes: heart murmur or valve disease; etiology with the groups of *Streptococcus mutans*, *Streptococcus bovis*, *Streptococcus sanguinis*, or *Streptococcus anginosus*; number of positive blood cultures ≥2; duration of symptoms ≥7 days; only 1 species growing in blood cultures; and community-acquired infection [8].

^g^n = 194, the 16 patients who died during index hospital admission were excluded from recurrence, duration of therapy end point, and management of central line analysis.

No patients in the cohort developed definite IE, and 1 patient with polymicrobial bacteremia had possible endocarditis due to coagulase-negative staphylococci. One hundred fifteen patients had a TTE (55%) within 7 days of BSI. Of these 115 patients, 1 had suspected valvular vegetations on TTE, but confirmatory TEE did not show any vegetations. Two patients had negative TTEs, but given a high clinical suspicion a subsequent cardiac CT was performed, which showed no valvular vegetations. No patient underwent positron emission tomography­–CT or single-photon emission-CT imaging. Despite negative cardiac imaging (TTE followed by cardiac CT), 1 patient completed empiric treatment for possible IE by Duke-ISCVID criteria [7]. The patient had 1 major criterion: *Staphylococcus schleiferi* bacteremia in 3 sets of blood cultures (drawn on 2 days); and 2 minor criteria: fever and vascular phenomena (possible splenic abscesses). The patient had a single blood culture bottle that also grew *S. mitis* concurrent with *S. schleiferi*. Therefore, the patient received a 6-week course of vancomycin therapy.

Recurrence of *S. mitis* BSI within 12 weeks of first positive blood culture occurred in only 3 patients. All had negative cardiac imaging upon documentation of recurrent infection. One patient recurred at 6.5 weeks after initial positive blood culture, presumptively from gastrointestinal translocation. A second patient with AML and metastatic colon cancer recurred 10.5 weeks after initial positive blood culture and was treated with 6 weeks of ertapenem for presumed liver abscesses related to colon cancer. The third patient recurred at 11 weeks and had concomitant pulmonary nodules and was treated with a course of empirical antifungal therapy and 4 weeks of IV ceftriaxone.

## DISCUSSION

Previous studies [5, 6] suggest that alpha hemolytic streptococcal BSI is rarely linked to IE in patients with neutropenia, but these were limited by lack of both cardiac imaging and description of the clinical syndrome. Others have hypothesized that the risk for endocarditis is lower in this population due to thrombocytopenia, which is often concurrent with neutropenia in patients with HM, as platelets play a key role in the pathogenesis of vegetation formation [6]. In this cohort of patients with concurrent neutropenia and *S. mitis* BSI, most patients had an acute febrile illness without longer-term fevers that might suggest subacute IE. Fifty-five percent of patients underwent cardiac imaging, and no patients developed definitive IE based on cardiac imaging and application of updated Duke-ISCVID criteria. These results suggest that obtaining cardiac imaging in this patient population solely based on the presence of *S. mitis* BSI is likely unnecessary.

Ninety-five patients did not undergo cardiac imaging. To ensure that a diagnosis of IE was not inadvertently missed, we assessed for recurrent *S. mitis* BSI within 12 weeks. Three patients had recurrence after initial therapy, 2 of whom did not undergo TTE initially. However, cardiac imaging with subsequent BSI was not consistent with IE, suggesting that the recurrence within a 12-week period was likely not related to undiagnosed IE. Despite negative TTE and cardiac CT, 1 patient received 6 weeks of empiric vancomycin therapy for concurrent *S. schleiferi* and possible IE. Not surprisingly, many patients with a fluoroquinolone-resistant *S. mitis* isolate were exposed to a fluoroquinolone within the previous 90 days. Although use of fluoroquinolone prophylaxis during neutropenia is recommended by guidelines [9, 10], prior exposure can limit oral treatment options for patients who develop *S. mitis* BSI.

This study is limited by the low number of patients with underlying cardiac valvular pathology, previous IE, mechanical valves, or cardiac devices that might predispose to endocarditis. It is not clear based on our study whether neutropenic patients with *S. mitis* BSI with underlying valvular disease/pathology are at greater risk for IE and therefore may need cardiac imaging performed. Additionally, because the median duration of therapy was about 2 weeks, it is possible that there were some undiagnosed cases of IE, particularly right-sided IE, cured by 2 weeks of antibiotics.

In conclusion, *S. mitis* BSI is a relatively common acute febrile illness in chemotherapy-induced neutropenia, likely the result of transient translocation of organisms from damaged mucosal surfaces, and was not a significant cause of IE in our cohort. Routine use of cardiac imaging to rule out IE appears unnecessary for many patients in this population.

## Notes

***Patient consent*.** This study does not include factors necessitating patient consent.
